# Increase in non-professional phagocytosis during the progression of cell cycle

**DOI:** 10.1371/journal.pone.0246402

**Published:** 2021-02-05

**Authors:** Alexander Hofmann, Florian Putz, Maike Büttner-Herold, Markus Hecht, Rainer Fietkau, Luitpold V. Distel

**Affiliations:** 1 Department of Radiation Oncology, Universitätsklinikum Erlangen, Friedrich-Alexander-Universität Erlangen-Nürnberg, Erlangen, Germany; 2 Department of Nephropathology, Institute of Pathology, Universitätsklinikum Erlangen, Friedrich-Alexander-Universität Erlangen-Nürnberg, Erlangen, Germany; Institut de Genetique et Developpement de Rennes, FRANCE

## Abstract

Homotypic or heterotypic internalization of another, either living or necrotic cell is currently in the center of research interest. The active invasion of a living cell called entosis and cannibalism of cells by rapidly proliferating cancers are prominent examples. Additionally, normal healthy tissue cells are capable of non-professional phagocytosis. This project studied the relationship between non-professional phagocytosis, individual proliferation and cell cycle progression. Three mesenchymal and two epithelial normal tissue cell lines were studied for homotypic non-professional phagocytosis. Homotypic dead cells were co-incubated with adherent growing living cell layers. Living cells were synchronized by mitotic shake-off as well as Aphidicolin-treatment and phagocytotic activity was analyzed by immunostaining. Cell cycle phases were evaluated by flow cytometry. Mesenchymal and epithelial normal tissue cells were capable of internalizing dead cells. Epithelial cells had much higher non-professional phagocytotic rates than mesenchymal cells. Cells throughout the entire cell cycle were able to phagocytose. The phagocytotic rate significantly increased with progressing cell cycle phases. Mitotic cells regularly phagocytosed dead cells, this was verified by Nocodazole and Colcemid treatment. Taken together, our findings indicate the ability of human tissue cells to phagocytose necrotic neighboring cells in confluent cell layers. The origin of the cell line influences the rate of cell-in-cell structure formation. The higher cell-in-cell structure rates during cell cycle progression might be influenced by cytoskeletal reorganization during this period or indicate an evolutionary anchorage of the process. Recycling of nutrients during cell growth might also be an explanation.

## 1 Introduction

In recent years more and more attention has been paid to non-professional cell phagocytosis [1–8]. Non-professional phagocytosis describes the mechanism of cell phagocytosis by ordinary epithelial or mesenchymal cells [3]. This phenomenon was mostly observed in cancer and has been termed cell cannibalism [9]. Cannibalism implies that the cancer cell has an advantage by phagocytizing other cells, however the prognostic significance of cell cannibalism in studies has been mixed and a favourable as well as an unfavourable association with prognosis has been observed [10]. An astonishing mechanism in the spectrum of non-professional phagocytosis is entosis, a process in which a living cell actively invades another living cell thereby forming a cell-in-cell structure (CIC) [11]. In the majority, these internalized cells undergo cell death. However, cells can divide inside the host cell and even escape from the outer cell [9].

The engulfment of dead cells can be regarded as an opposing process. Here the host cell participates as the active part and engulfs a dead necrotic or apoptotic cell [12]. This mechanism may be physiologic with the goal to eliminate dying cells from tissue to prevent inflammation and severe damage. We could show that a broad range of 21 different normal tissue cells derived from seven different organs are all capable of non-professional phagocytosis. In skin tissue and around the infarction zone of myocardium after a myocardial infarction cell-in-cell structures have been demonstrated [13]. Yet in tissue it is not possible to differentiate between entosis and non-professional phagocytosis of dead cells.

In previous studies, we were very surprised to frequently observe mitotic normal tissue cells being involved in cell-in-cell structure formation. In the present study, we therefore aimed to explore the relationship of cell cycle phase and the propensity for non-professional phagocytosis.

## 2 Material and methods

### 2.1 Cell lines and cultivation

The capability of adherent cells to act as non-professional phagocytes was studied using five different human cell lines. Normal human bronchial epithelium cells (BEAS-2B) were obtained from the United Kingdom Sigma/Public Health Consortium. Infection of this epithelial-like growing cell line with adenovirus type 12 (Ad12) and Simian virus 40 (SV40) hybrid virus has led to unlimited proliferative potential [14, 15]. HEK293 (CRL-1573, ATCC) cell line was derived by immortalising human embryonic kidney cells with sheared adenovirus type 5 (Ad5) DNA [16, 17]. Aforementioned cell lines were cultivated in Dulbecco´s modified eagle´s medium (DMEM, Pan-biotech, Life Technologies, Darmstadt, Germany), 10% foetal bovine serum (FBS-Superior, Biochrom GmbH, Berlin, Germany) and 1% penicillin/streptomycin antibiotics medium (Life Technologies GmbH, Darmstadt, Germany). Human Tenon’s capsule fibroblasts (HTKF) were isolated from normal donor eyes. Eyes were dissected and digested by collagenase followed by centrifugation. The primary fibroblast cell lines SBLF7 and TE were isolated from human Caucasian donors. Skin biopsies were taken and cut into very small pieces and culture in flasks. Emigrating fibroblasts were cultivated. Fibroblasts were cultured in F12-Medium (Life Technologies GmbH, Darmstadt, Germany). Medium was supplemented by 15% foetal bovine serum, 5% non-essential amino acids (Life Technologies, Darmstadt, Germany) and 1% penicillin/streptomycin antibiotics medium (Life Technologies GmbH, Darmstadt, Germany).

### 2.2 CIC induction in adherent cells

75,000 cells were seeded on a coverslip and incubated at 37°C for 48 hours to grow to a confluent cell layer. 150,000 homotypic living cells were stained red using CyTRAK Orange (Abcam, Cambridge, UK). Subsequently, necrosis was induced by a hyperthermic (56°C) bath for the duration of one hour. Viable and necrotic cells were co-incubated for 1, 2 and 4 hours (37°C, 5% CO_2_), respectively.

### 2.3 G2/M arrest by Nocodazole/Colcemid

Cells were seeded on a coverslip as described above and after growing to a cell layer treated with either Nocodazole (100μM) or Colcemid (0.3 μg/ml) for 16 hours. In one run medium containing the reagents was washed out and green stained (CTOG) necrotic cells were added for the duration of 2 hours. Furthermore, necrotic cells were added to the medium of cell layers still including Nocodazole or Colcemid and coincubated for 6 hours.

### 2.4 Mitotic shake-off and cell cycle synchronization by Aphidicolin

Mitotic shake-off was used to generate mitotic cells for cell cycle phase specific CIC rate studies. 3–5 x 10^6^ cells were cultured in T150 flasks and incubated for 48 hours (37°C, 5% CO_2_) to increase cell numbers. Cell culture flasks were vortexed manually at 1600–1800 rpm on a vortex mixer. Mitotic cells were detached from the flasks due to the lower adhesion to the flask. The medium containing the mitotic cells was collected and held on ice. Cells were synchronized to yield high amounts of cells in G1 and S phase. The specific DNA polymerase α inhibitor Aphidicolin (1 μg/ml) was used to arrest cells in G1/S. Aphidicolin was washed out and cells were cultured for 1, 3, 6 and 10 hours to enrich cells in early S, S, late S and G2 phase, respectively. For each cell cycle phase two different samples were generated. On the one hand, 75,000 cells were seeded on a coverslip to adhere. These cells were co-incubated with red-stained necrotic cells for one hour. On the other hand, 75,000 cells of the same underlying population of cells were fixed with 70% ethanol and subsequently stained with Hoechst 33342 for cell cycle analysis via flow cytometry measurement. The cell cycle time of the studied lung cells (BEAS) was expected to be 16 h.

### 2.5 Fluorescence staining

The coverslips were fixed in 4% formaldehyde solution and blocked with blocking buffer (4% FCS, 0.3% Triton X-100 in phosphate-buffered saline) at 4°C overnight. Afterwards slides were washed in phosphate-buffered saline (3 x 5min) and incubated with primary antibodies overnight at +4°C in a humidified chamber. Primary antibody solution contained 0.01g/ml BSA and 0.03% Triton X-100 in PBS as well as Ki-67 (1:50, mouse, Santa Cruz Biotechnology, Inc.) and alpha-tubulin (1:250, rabbit, Abcam, Cambridge, UK). After one more washing step the coverslips were incubated at room temperature in a humidified chamber with secondary antibodies goat anti-Mouse-Alexa 488 (1:400, A11001, Life Technologies) and goat anti-Rabbit-Alexa 647 (1:200, goat, Life Technologies) for 90 min. Coverslips were washed and dried and mounted on glass slides using ProLong^™^ Gold Antifade Mountant reagent (Thermofisher Scientific). The mounting medium contained DAPI for nuclear fluorescence staining.

### 2.6 Imaging and image analysis

Images were acquired using a semi-automatic microscope (Axio Imager Z2, Carl Zeiss Microscopy, Göttingen, Germany) at 400x or 630x magnification. Manually adjusted areas were scanned and afterwards analysed with Biomas image-processing software (MSAB, Erlangen, Germany). The number of observed cell-in-cell structures as a fraction of all viable cells was defined as CIC rate. Statistical analysis was performed with Graphpad Prism 8 (GraphPad, San Diego, CA, USA). Mann-Witney-U-Test was used for statistical testing.

### 2.7 Criteria for CIC evaluation

**Fig 1:**
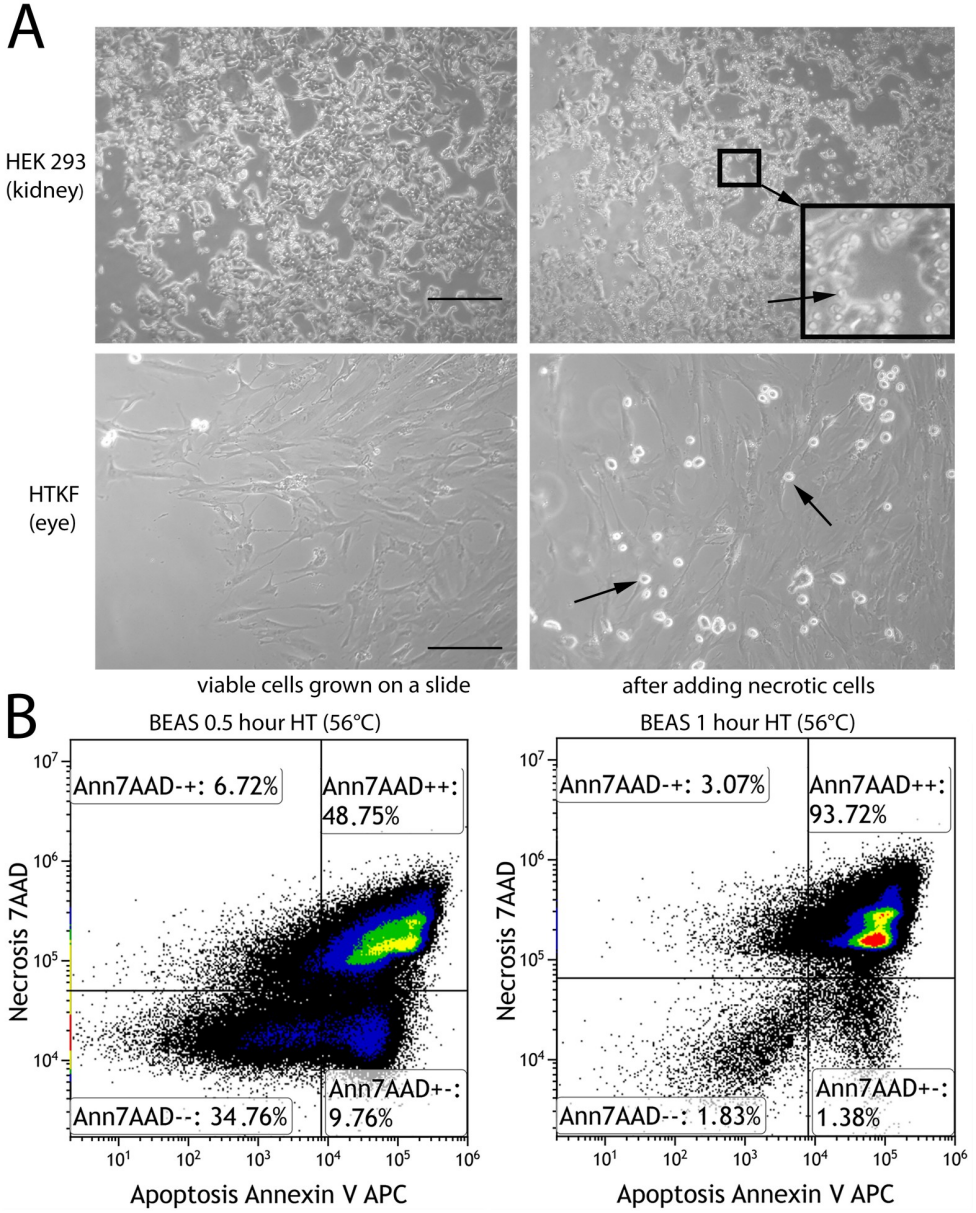
Induction of non-professional phagocytosis by hyperthermia. (A) Microscopic images (100x magnification) of kidney cells and human Tenon’s capsule fibroblasts grown in coherent cell layers prior to and after adding necrotic, homotypic cells. Arrows are pointing at round shaped necrotic cells. Scale bars are 100μm. (B) Flow cytometric analysis of human bronchial epithelium cells (BEAS) after hyperthermia treatment for half an hour and one hour duration. Annexin V-APC and 7-amino-actinomycin D were used to detect apoptotic and necrotic cells, respectively.

A detected cell structure had to fulfil the following criteria to be counted as a CIC: The nucleus of the viable host cell is crescent-shaped due to compression by the phagocytised cell and has increased chromatin density at the semi-circular point of contact. The cellular cytoskeleton, represented by alpha-tubulin, is arranged circularly around the internalised necrotic cell. The incorporated cell displays a DNA containing nucleus and approximately spherical shape due to the lack of cellular junctions (Fig 1A).

## 3 Results

### 3.1 Cell-in-cell structures in adherent cell layers

**Fig 2:**
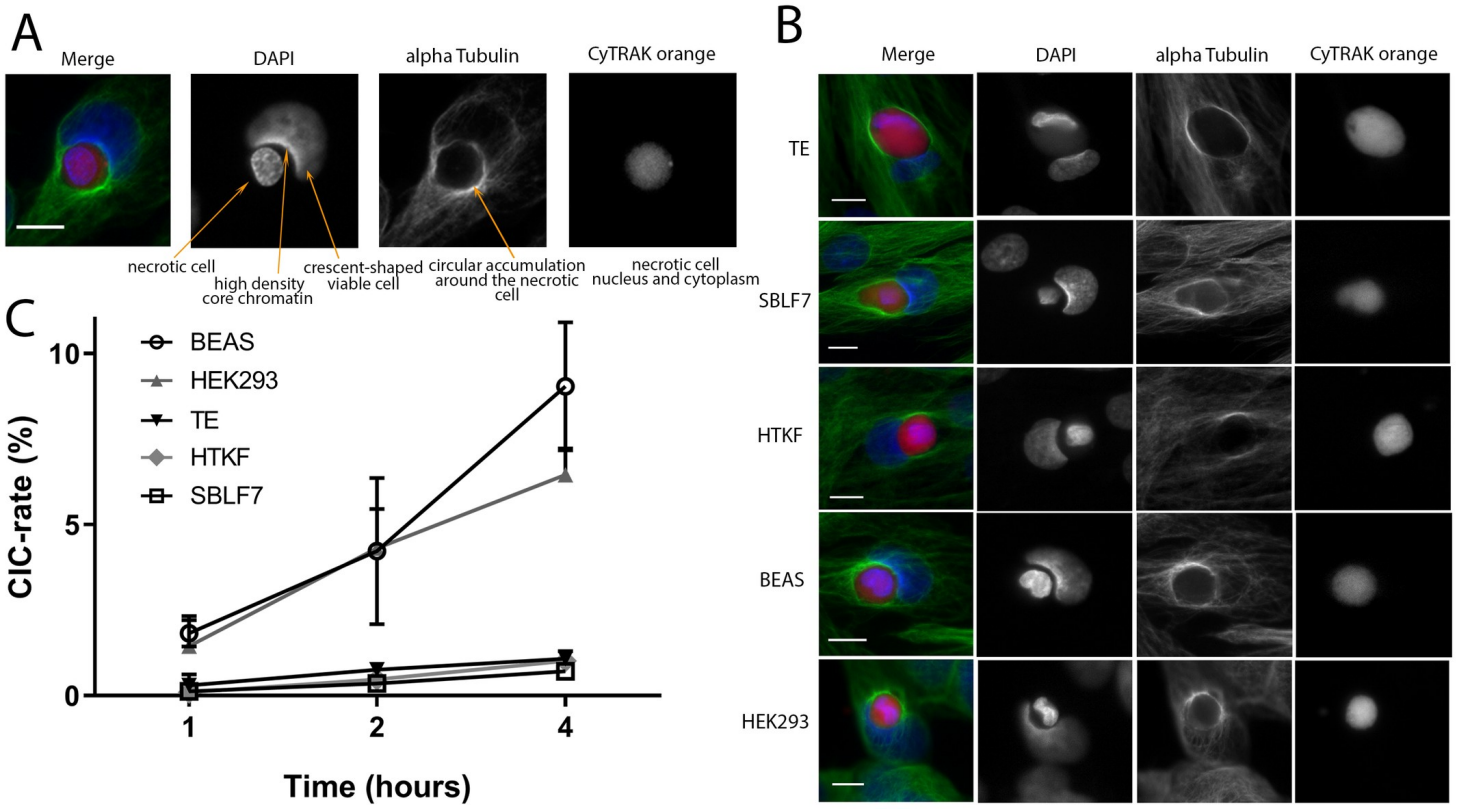
Non-professional phagocytosis in mesenchymal and epithelial cells. (A) Criteria for CIC evaluation are examplarily shown for a human bronchial epithelial cell (BEAS). Nuclei staining is performed with DAPI (blue), cytoskeleton with alpha-tubulin (green) and necrotic cell with CyTRAK orange (red). (B) CIC formation in the studied cell lines in confluently grown cell layers. Scale bars indicate 10 μm (C) Non-professional phagocytosis rates of the five studied cell lines are calculated as a ratio of observed cell-in-cell structures to all detected viable cells. All experiments were repeated at least three times and not less than 1000 viable cells were counted.

Non-professional phagocytosis was studied in cells of epithelial and mesenchymal origin growing in an adherent cell layer (Fig 1A). One fraction of the cells was heated up to 56°C for 1 hour and mostly became necrotic (Fig 1B). The remainder of the cells grown on a slide and coincubated with red-stained, heat-treated necrotic cells formed typical cell-in-cell structures with a crescent-shaped nucleus of the host cell and a round shape of the red cell completely engulfed by the cytoskeleton of the host cell (Fig 2A). All studied cell lines generated cell-in-cell structures. The non-professional phagocytic process is depicted for all five cell lines. Showing different stages of phagocytosis, the TE cell line just begins to fuse and the SBLF7, HTKF and BEAS cell lines are continuing the process of phagocytosis. In HEK293 the dead cell is completely enclosed and is probably already being degraded (Fig 2B). Immortalized human bronchial epithelial cells (BEAS) and the human embryonic kidney cells (HEK293) phagocytized a distinctly higher number of cells after 2 and 4 hours of incubation than human skin and eye fibroblasts (TE, SBLF7 and HTKF) (Fig 2C). BEAS appeared to be the most active phagocytizing cell line as almost 1 of 10 viable cells engulfed a necrotic cell 4 hours after co-incubation. Phagocytosis rates rose with increasing co-incubation period for each studied cell line. Phagocytosis started early having engulfed 0.09% of human capsule fibroblasts (HTKF) after one hour, 0.48% after two hours and 1.01% after 4 hours. CIC frequencies of skin fibroblasts (TE, SBLF7) ranged from 0.12% to 1.08%.

**Fig 3:**
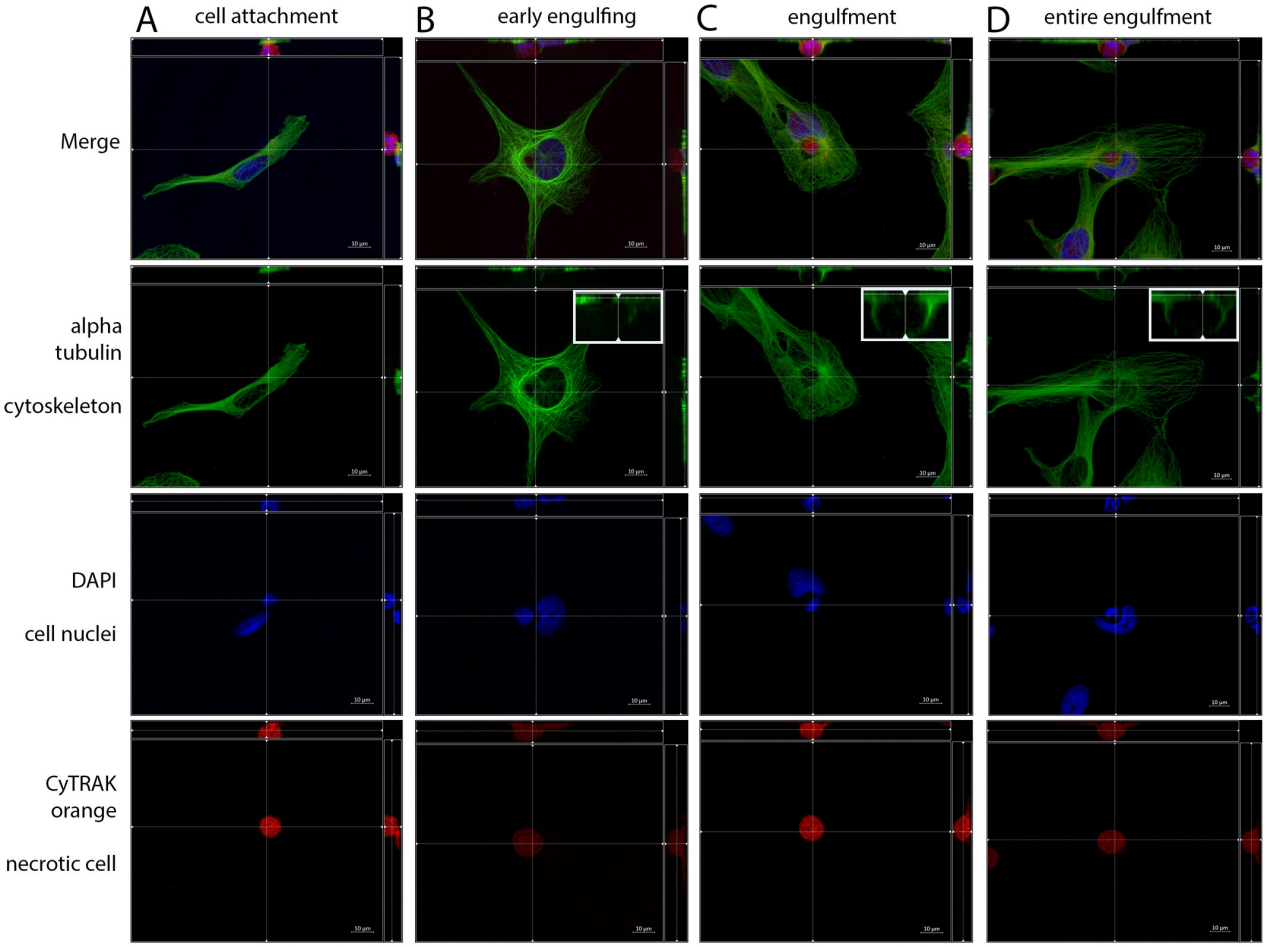
Different phases of non-professional phagocytosis. Four different phases of cell-in-cell structure formation. α-tubulin was used for cytoskeleton staining, DAPI for cell nuclei and CyTRAK orange for necrotic cell staining. Using the microscope’s Z-stack imaging sequence enabled a representation in XYZ planes. (A) Cell attachment, (B) early engulfing cell, (C) engulfment and (D) late entire engulfment of the dead (red) cell by the living cell (green). Enlarged sections display the reconstruction of α-tubulin cytoskeleton around the necrotic cell.

Progressive crescent-shaped deformation of the non-professional phagocytes nucleus and changes of the microtubular cytoskeleton were observed both within the different cell lines studied and within a specific cell line. The phase of non-professional phagocytosis could be determined by assessing the distance between the nuclei, the degree of deformation and the spatial distribution of the alpha-tubulin cytoskeleton. At the beginning of the engulfment, there was no deformation of the non-professional phagocyte’s nucleus and the whole cytoskeleton was attached to the coverslip (Fig 3A). Phagocytosis started by reorganization of the cytoskeleton around the necrotic cell by developing a junction between the viable and necrotic cell. A progressive deformation of the nucleus begins (Fig 3B). Reflecting the progress of enclosing the necrotic cell, alpha-tubulin forms a dome around the necrotic cell for complete inclusion (Fig 3C). The necrotic cell was completely engulfed and the nonprofessional phagocyte’s nucleus obtained a marked crescent shape (Fig 3D). In overview scans of the five cell layers major differences were observed in the number of mitotic cells between the cell lines. We therefore studied the respective proliferation rates via Ki-67 labelling addressing the question of whether cell proliferation and non-professional phagocytosis are associated.

### 3.2 Proliferation rates of the analyzed cell lines

**Fig 4:**
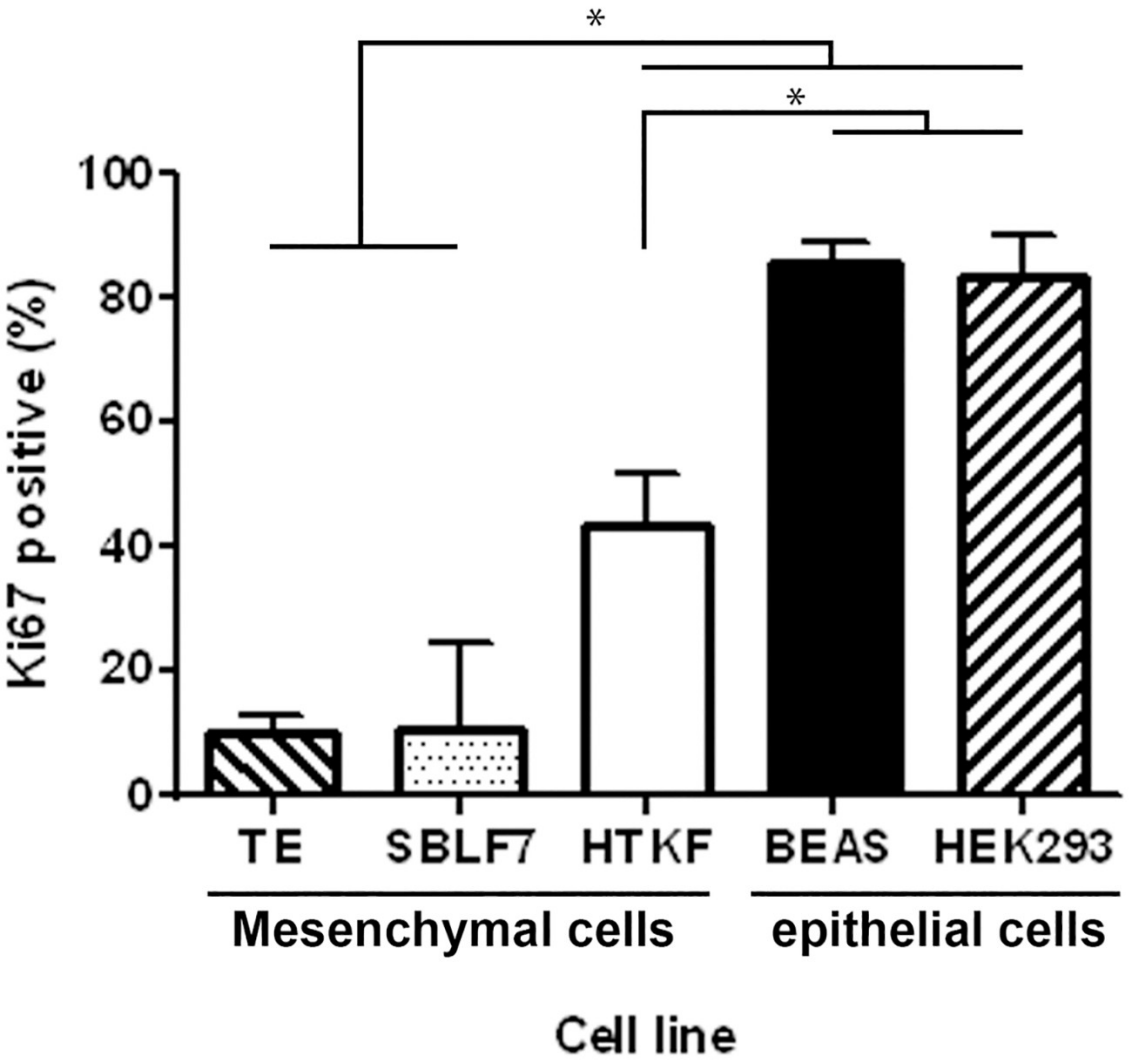
Proliferation rates of mesenchymal and epithelial cells. Ki-67 labelling index of the five healthy human tissue cell lines. Proliferation rates were calculated as fraction of Ki-67 positive of all nuclei. All experiments were repeated at least four times (two-sided Mann–Whitney U; *p < 0.05).

Cell line specific proliferation rates were studied by Ki-67 labelling index. Immunofluorescence staining was performed for adherently grown cell layers of above-mentioned healthy human tissue cell lines. Human bronchial epithelial cells (BEAS) and the human embryonic kidney cells (HEK293) had clearly higher rates of nuclei stained positive for Ki-67 than the studied fibroblast cell lines (Fig 4). The lung cells’ proliferation rate of 85.5% was the highest among all analysed cell lines, whereas 10.1% in TE primary fibroblasts represented the lowest rates. These findings were similar to the observed differences in CIC formation and thus suggested an association between high CIC rates and Ki-67 labelling index.

### 3.3 Influence of decreased growth factor stimulation on non-professional phagocytosis

**Fig 5:**
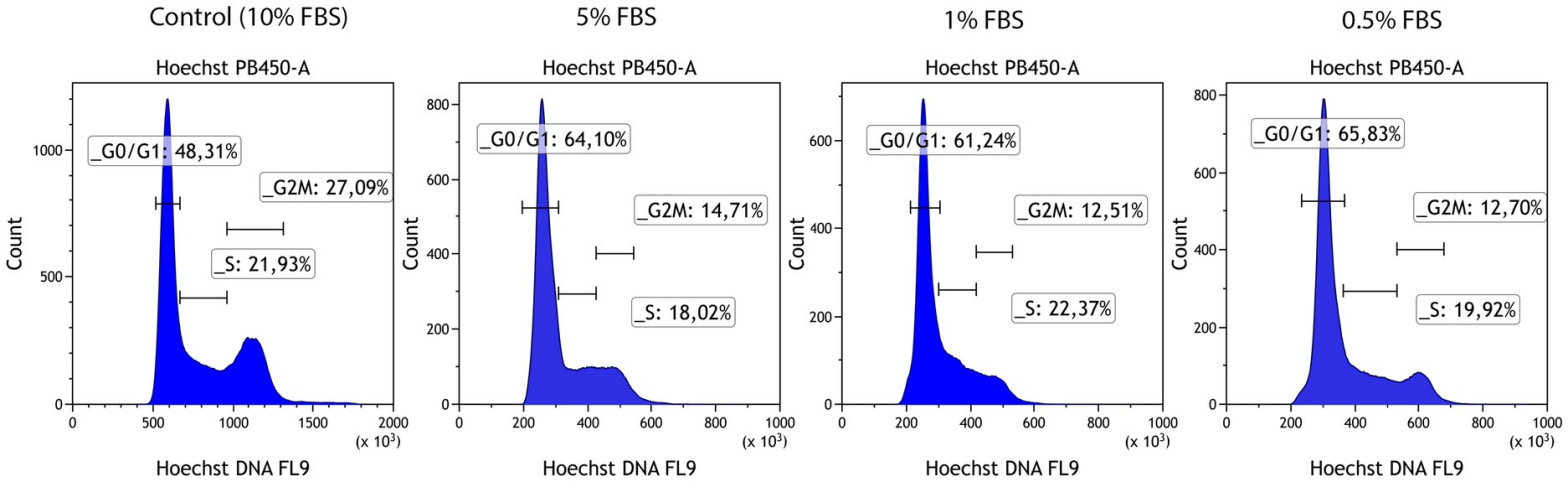
Cell cycle distribution after serum deprivation. Flow cytometric analysis of different cell-cycle phases using Hoechst 33342 staining after two weeks of cultivation in medium supplemented with fetal bovin serum at concentrations of 0.5%, 1%, 5% and 10%, respectively.

The extremely high proliferation rates of the HEK293 and BEAS cell lines and the coincidently high cell-in-cell formation rates raised the question, whether cells entering the cell cycle are actively participating in non-professional phagocytosis. We downregulated the proliferation rate of human epithelial lung cells by reducing the content of FBS in cell culture medium. Cells were treated with medium containing 5%, 1% and 0.5% FBS for two weeks, respectively. DNA content analysis was performed by Hoechst staining and flow cytometry. The non-professional phagocytosis rate was estimated by the above-mentioned method. Most cells were in G0/G1 cell cycle phases (Fig 5) and CIC rates did not vary clearly between cells receiving 10% FBS in cell culture medium and cells with reduced FBS content (5%, 1%, 0.5%). Next, we hypothesized that possibly cells in S-phase or G2/M-phase could be responsible for the increased rate of non-professional phagocytosis observed in rapidly proliferating cell lines.

### 3.4 Phagocytosis occurs at all stages of the cell cycle and is most prominent in the G2/M phase

**Fig 6:**
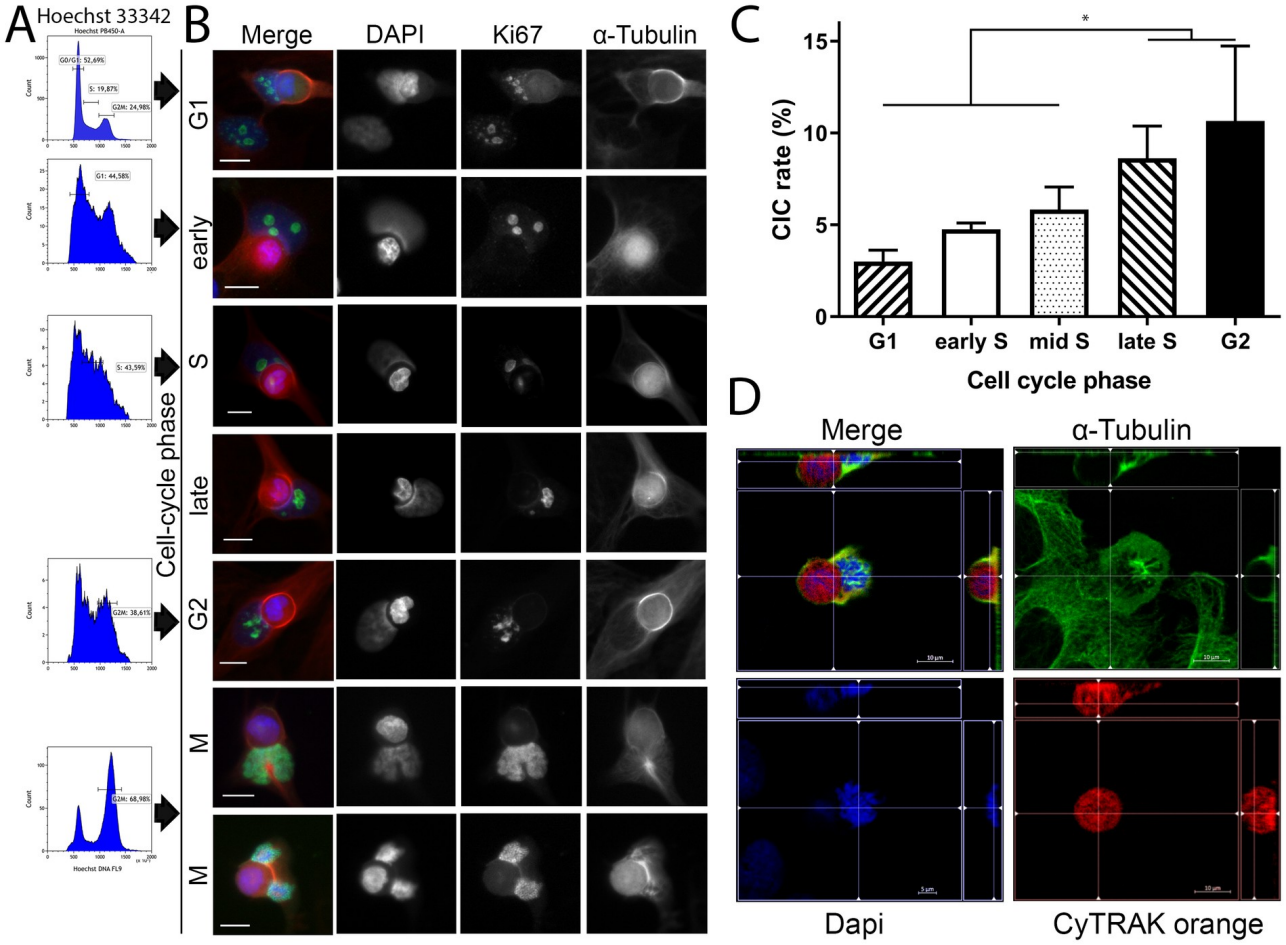
Frequency of non-professional phagocytosis as a function of cell cycle phases. (A) Flow cytometric analysis of different cell-cycle phases with Hoechst 33342 staining. The histograms depict the distribution within the cell cycle of a synchronus BEAS cell population. (B) Fluorescence microscopy images of cell-in-cell structures from various cell-cycle phases. Cell nuclei staining was performed by DAPI, Ki-67 served as proliferation marker and α-tubulin was used for cytoskeletal staining. Scale bars: 5μm. (C) Cell cycle phase specific uptake rates of human bronchial epithelial cells (BEAS). All experiments were repeated at least four times. (two-sided Mann–Whitney U; *p < 0.05) (D) Microscopic image in three dimensions of a mitotic cell incorporating a necrotic homotypic cell. Different planes were selected in the four images. The necrotic cell is completely encircled by the non-professional phagocyte’s alpha-tubulin cytosceleton.

BEAS cells phagocytosed the highest amount of cells and had a very high proliferation rate. Therefore, we studied whether cell-cycle phase affects the propensity of cells to perform non-professional phagocytosis. Mitotic shake-off was used to generate mitotic cells with subsequent treatment using the DNA polymerase α inhibitor Aphidicolin for synchronizing cells at the G1/S border. Approximately 70% of shaken-off cells had G2/M specific DNA content. The efficiency of generating synchronized cells was confirmed by flow cytometric analysis of Hoechst stained cell nuclei (Fig 6A). Adherent viable cells at different cell cycle phases were coincubated with red-stained necrotic cells for 1 hour.

**Fig 7:**
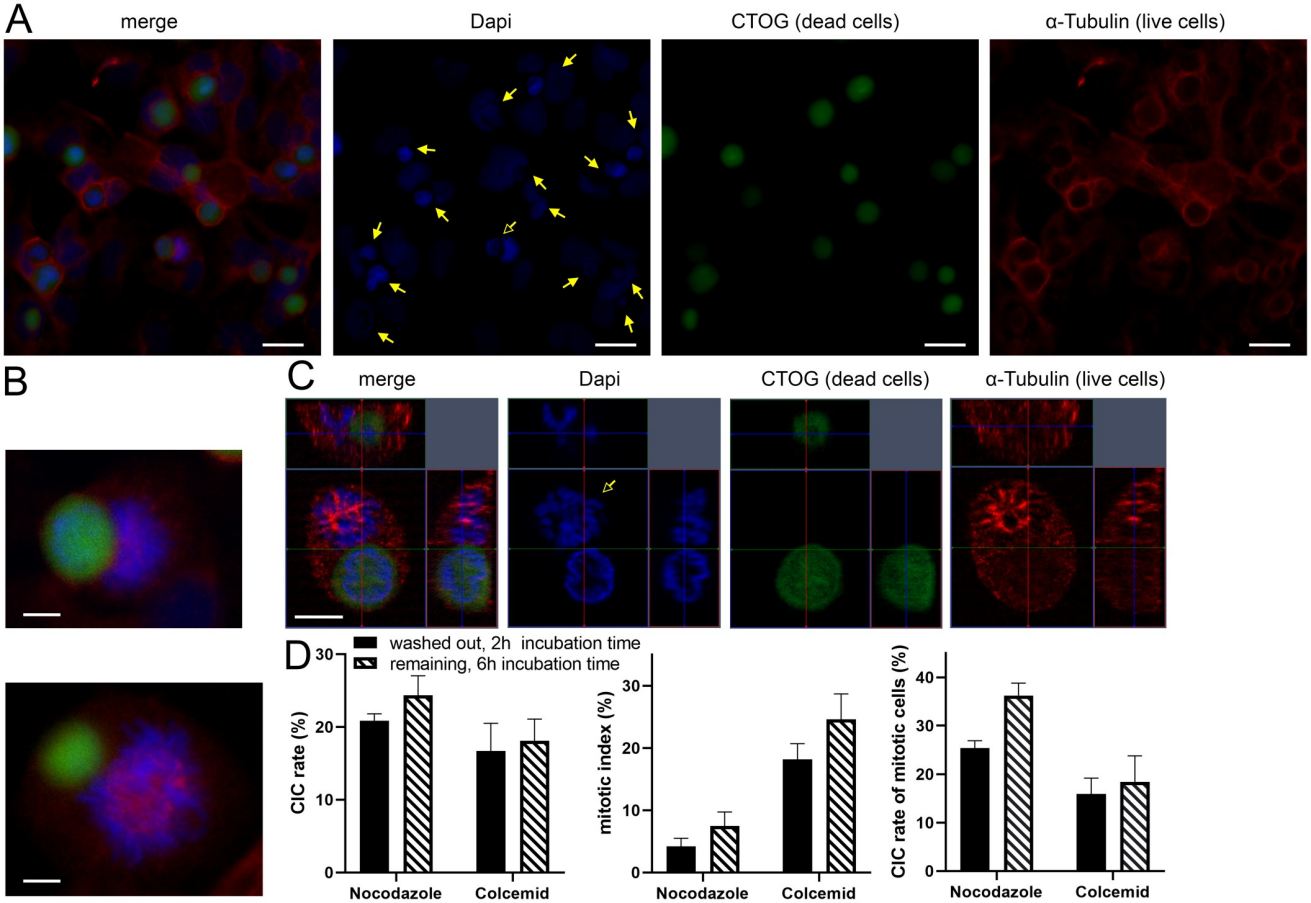
Nocodazole and Colcemid increase mitotic CIC rates in BEAS. (A) Overview scan of a human lung cell layer treated with Nocodazole for 16 hours. DAPI was used for cell nuclei staining, CTOG for necrotic cells and α-tubulin for cytoskeleton. Filled arrows indicate CIC-structures, unfilled arrows points to mitotic cell involved in non-professional phagocytosis. Scale bars: 10μm. (B) Mitotic cells involved in non-professional phagocytosis. Scale bars: 5μm. (C) Three-dimensional microscopic image of a mitotic cell that has completely absorbed a necrotic cell in its cytoplasm. Scale bars: 5μm. (D) The left bar chart maps CIC-rates of BEAS after treatment either with Nocodazole or Colcemid for 16 hours. Middle: mitotic index of lung cells, which was calculated as the proportion of mitotic cells in all living cells. Right bar chart: Mitotic cells involved in CICs, calculated as proportion of mitotic cells that have incorporated a necrotic cell compared to all mitotic cells.

Cells in any phase of the cell cycle were able to phagocyte necrotic cells (Figs 6B and 7). However intriguingly, with cell cycle progression from G1 to G2 phase an increasing number of CIC structures was observed. Cells in G1 had the lowest CIC rates (3.0%) that increased in S-phase and reached a maximum of CICs in G2 phase (10.6%) (Fig 6C). Even mitotic cells were able to engulf necrotic cells in our assay (Fig 6), although quantification of non-professional phagocytes in mitosis was precluded by the short time of the mitotic phase and the missing adherence of mitotic cells. By enabling a three-dimensional depiction of the cytoskeleton, microscopic Z-stack imaging demonstrated complete encirclement of the internalised cell with DAPI nuclear staining demonstrating condensed DNA chromosomes of a mitotic cell (Fig 6D, S1 and S2 Videos). Next we verified that mitotic cells phagocytise dead cells using drugs that interfere with the microtubules to obtain cells in G2 and the mitosis phase.

### 3.5 Mitotic cells are regularly involved in non-professional phagocytosis

**Fig 8:**
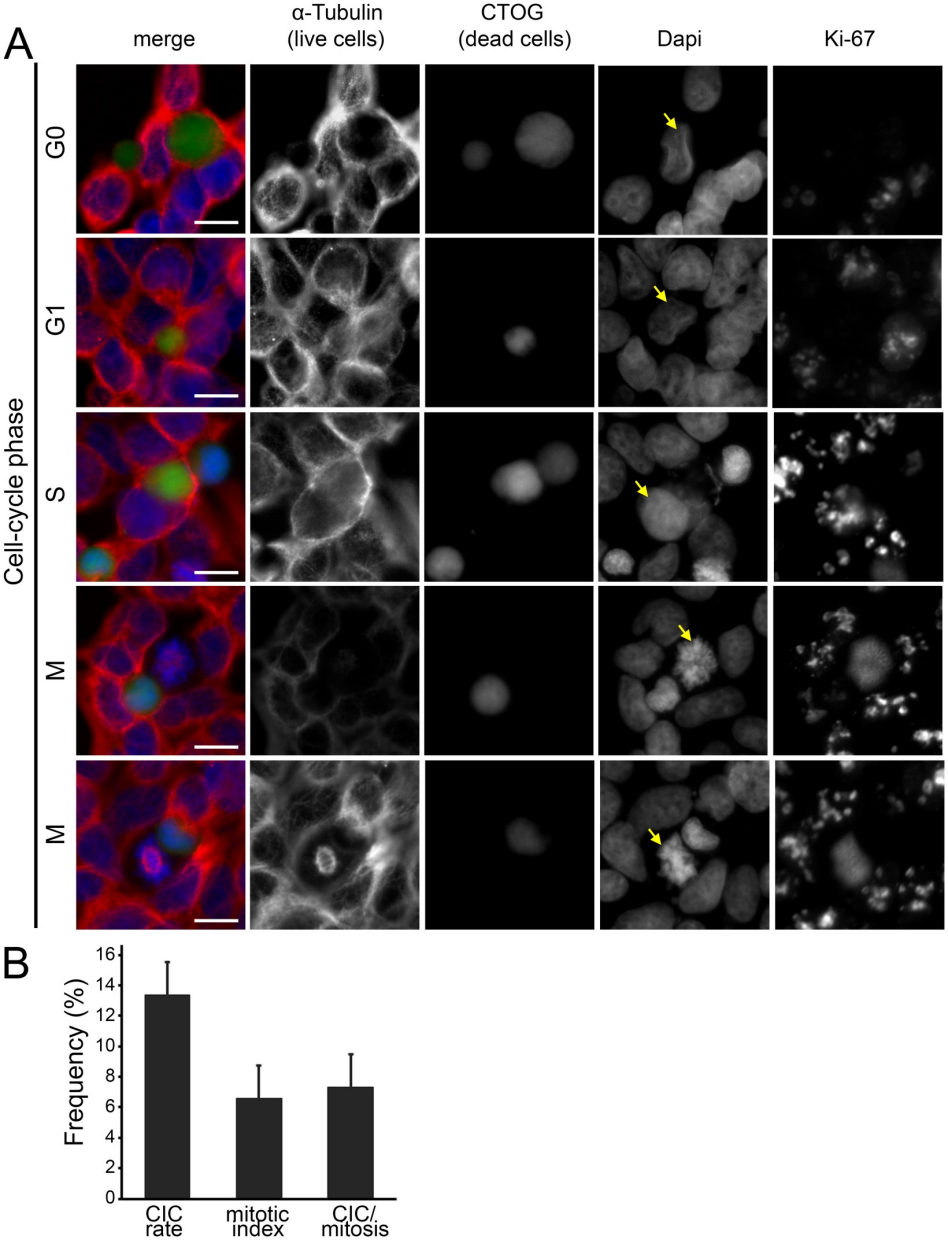
CICs as a function of all cell cycle phases in HEK293 cells. (A) Fluorescence microscopy images of cell-in-cell structures from various cell-cycle phases. Cell nuclei staining was performed by DAPI, α-tubulin was used for cytoskeletal staining, dead cells were stained by CTOG and Ki-67 served as proliferation marker. Ki-67 was not included in the merged image. Scale bars: 5μm. (B) CIC-rates, mitotic index and mitotic cells involved in CICs of HEK293 cells after treatment with Nocodazole for 16 hours.

**Fig 9:**
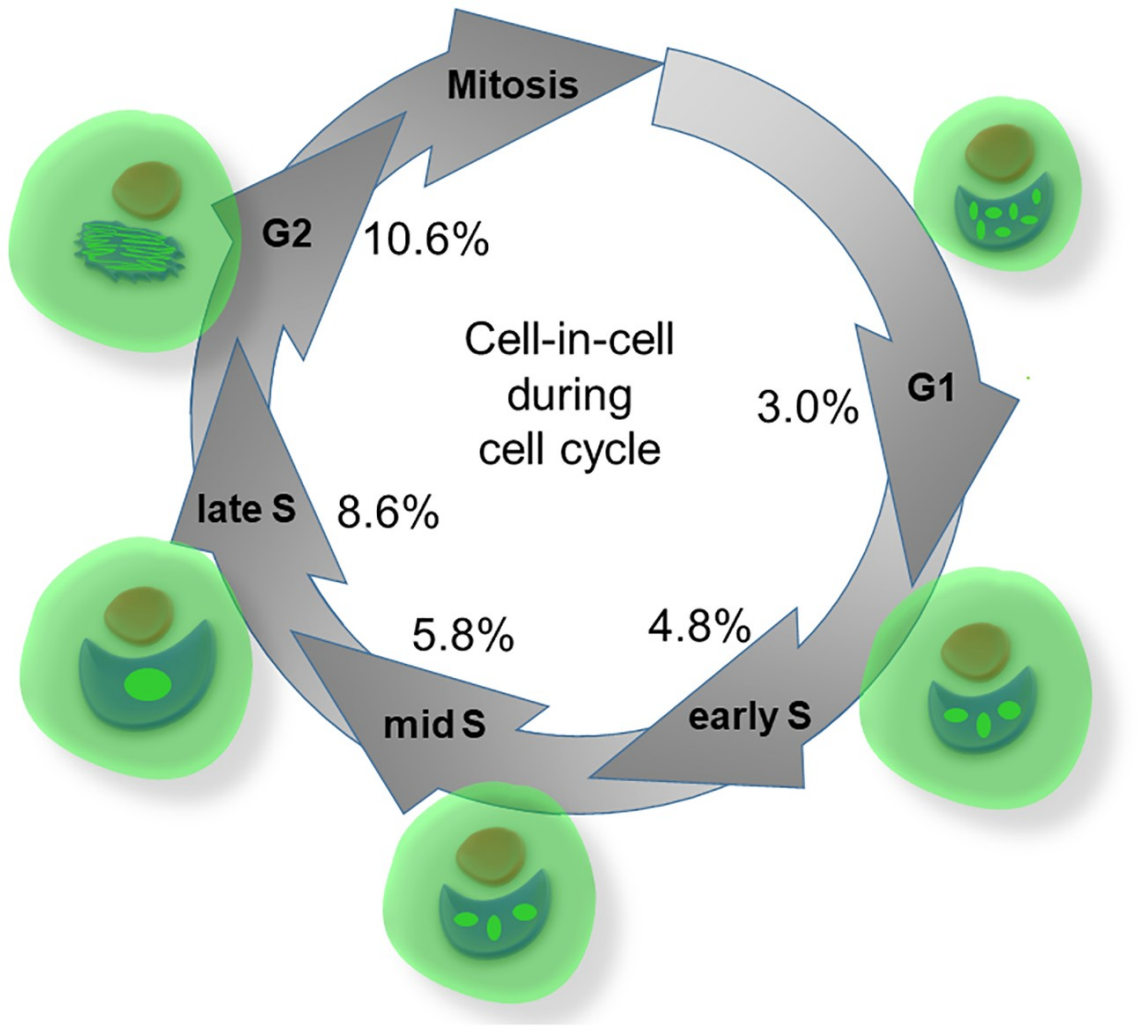
Schematic illustration of CIC rates during the progressing cell cycle. Schematic illustration of cell cycle phases and specific engulfment rates by BEAS cells. Ki67 as proliferation marker shows specific staining pattern.

Analysis of bronchial epithelial cells (BEAS) indicate an increase in CIC structures after treatment with Nocodazole (Fig 7A). This drug chemically interferes with the microtubule organization in the cells and arrests them in G2/M. Furthermore, an increased number of mitoses in which a necrotic cell was incorporated could be observed (Fig 7B and 7C, S1 Fig). Compared to previous analyses, an increased rate of CIC was found both Nocodazole (24.4% / 20.8%) or Colcemid (18.1% / 16.7%) in the medium and after washout. The CIC rates of cells treated with Nocodazole were higher than those of cells treated with Colcemid (p = 0.05 / p = 0.05). Next, we determined the mitotic index of the cells after drug treatment. The proportion of mitotic cells compared to all living cells was 4.2 percent after treatment with Nocodazole and 18.1 percent after treatment with Colcemid. To find out to what extent mitotic cells are involved in the uptake of necrotic cells, we determined the CIC rate of mitotic cells. After treatment with Nocodazole, a phagocytosis rate of mitotic cells of 25.5 percent could be detected after washing out the substances. With Colcemid the rates were comparatively lower (15.7%) (p = 0.05). Correspondingly, the phagocytosis rates were slightly higher with longer exposure time of the substances in the medium and longer incubation time (Fig 7D) (p = 0.05). Similar to BEAS cells, we found that HEK293 cells phagocytise in all phases of the cell cycle (Fig 8A). HEK293 cells had a lower CIC rate of 13.4% and a low mitotic index of 6.6% and 7.3% CIC per mitosis compared to the BEAS cells (Fig 8B). We have illustrated in a schematic figure the non-professional phagocytosis increasing with the progression of the cell cycle (Fig 9).

## 4 Discussion

The main finding of this study was that cells in all phases of the cell cycle are capable of non-professional phagocytosis. Even more astonishing was the fact that cells progressing in cell cycle up to G2 phase are even more active in phagocytic activity. The cell-in-cell rate rose from 3.0% in G0/G1 to 10.6% in G2 phase (Fig 9). Thus, cells in G2 were three times more active in forming cell-in-cell structures than G0/G1 cells. Additionally, even mitotic cells were able to phagocytose other dead cells. This point could be strengthened with the help of Nocodazol treatment and the associated increased rate of non-professional phagocytosis. Up to one third of the mitotic cells were able to accommodate a necrotic cell in their cytoplasm. During late G2 and early onset of mitosis the cell undergoes profound changes, including detachment from extracellular matrix and rearrangement of the cytoskeleton resulting in a spherical cell shape [18]. The physiologically increased plasticity of the cytoskeleton in this phase might be a plausible explanation for higher CIC rates during these cell cycle phases.

The increasing rate of CICs with cell cycle progression seems to be counterintuitive. We would have expected a decrease of CICs in cells that have entered the cell cycle, because there are high requirements for the complex tasks that must be performed. Especially in S phase the cell is focused on DNA synthesis. And even more during mitosis, in which the cell must divide and all cell components including chromosomes must be separated equally. Therefore, it is astonishing that cells in these sensitive phases phagocyte other dead cells. The reason for that may be that non-professional phagocytosis is a program deeply anchored in evolution [9]. Cell-in-cell structures were observed in bacteria [19, 20], amoebae [21], C. elegans and Drosophila elegans [22] illustrating the high conservation of this mechanism. However, this does not explain why cells phagocytose even more with cell cycle progression. An explanation for this could be that cells require a lot of essential nutrients for cell growth and use the phagocytized cells as reservoir of these materials. This would be similar to cell cannibalisms observed in cancers [10, 23–25]. Conversely, the high CIC rates in the G2 phase contradicts this theory, because although the synthesis of DNA and other cell components is completed, the CIC rates are even higher than in the S phase.

A possible explanation could be that cells use a similar mechanism as in entosis. Entosis is the process where an epithelial live cell is actively invaded by another live cell [11]. Mostly entosis was observed in detached cells in fluids [23, 26, 27]. Recently entosis was observed to occur among adherent epithelial cells [28, 29]. The essential step in forming entosis is the adhesion along the entire interface between both cells by adherens junctions consisting of cadherin and catenin [11]. A contractile zone consisting of actin and myosin II is formed and is regulated by Rho so that the invading cell is pushed into the host cell [9]. A recent study has identified mitosis as one of the triggers for entosis [28]. Here, mitosis was indispensable for cell-in-cell formation in adherent cells.

Due to the conserved process of phagocytosis similar mechanisms as in entosis may be involved in non-professional phagocytosis of dead cells. In contrary to entosis in our model we add dead cells to living cells so that mostly necrotic cells are engulfed by living cells [12]. However, both mechanisms result in a cell-in-cell structure. Even though the necrotic cell cannot actively invade a living cell and the engulfment must be initiated by the live cell. The subsisting cell has to initiate the phagocytizing process, probably with similar mechanisms as in the entotic process.

Entosis is exclusively observed in epithelial cells [11, 30]. We used adherent cells of mesenchymal and epithelial origin. Mesenchymal cells formed cell-in-cell structures at low rates and epithelial cells formed cell-in-cell structures to a much higher extent. Mesenchymal cells were capable of non-professional phagocytosis and this is a clear distinction to entosis. Resembling entosis, however, epithelial cells were phagocytizing at a much higher extent. An important finding was that phagocytosis activity increases as cells progress through the cell cycle with the highest rates observed in G2 phase. Even higher rates may occur in mitosis. Hitherto it was not possible to count mitotic cells, because the incubation phase is longer than the mitosis phase itself. In addition, mitotic cells are not adherent and thus it is difficult to quantify these events. Nonetheless it is an apparent similarity to entosis that mitotic cells have a high phagocytizing activity, however, with the main distinction being, that mitotic cells invade the host cell in entosis, while in our model the mitotic cell is the host cell and the dead cell is being engulfed. This may hint to the fact that similar mechanisms like Cdc42 downregulation and Rho activation are regulating phagocytosis in entosis and non-professional phagocytosis of necrotic cells.

We studied the capability of healthy human tissue cells to engulf homotypic necrotic cells in a confluently grown, adherent cell layer. We used normal tissue cell lines derived from healthy individuals to create a model close to physiological processes. Each studied cell line was able to phagocytose necrotic cells. Non-professional phagocytosis as common mechanism in normal tissue cells was already shown in an culture model for 21 different normal tissue cell lines from seven different organs [13]. The formation of a cell-in-cell structures appeared to be a fast process, because the first CIC structures between necrotic and viable cells formed just after one hour of coincubation.

## 5 Conclusion

Mesenchymal and epithelial normal tissue cells can phagocytize dead cells in vitro with epithelial cells exhibiting higher phagocytizing activity. The ability to phagocytize dead cells is maintained throughout the entire cell cycle. Contrary to all expectations, phagocytizing activity increased as the cell cycle progressed with even mitotic cells being able to engulf dead cells. These findings indicate an evolutionary strongly conserved process of non-professional phagocytosis.

## Supporting information

S1 Fig Mitotic cells phagozytizing dead cells. Cell nuclei staining was performed by DAPI, α-tubulin (red) was used for cytoskeletal staining, dead cells were stained by CTOG (green). (A) Orthogonal images of a dead cell which is not engulfed by an other cell depicting that this heat treaded cell has lost most of its α-tubulin. (B) Orthogonal images of an mitotic cell engulfing a dead cells. (C) Orthogonal images of mitotic cells engulfing dead cells.
(TIF)

S1 Video Mitotic BEAS cell has phagozytized a necrotic cell. A cell treated with Nocodazole for 16 hours. DAPI was used for cell nuclei staining, CTOG for necrotic cells and α-tubulin for cytoskeleton. Images were acquired by structured illumination (ApoTome) and video was created with the ZEN software.
(AVI)

S2 Video (AVI)
