# Supplementary figures and images for: Increase in non-professional phagocytosis during the progression of cell cycle
Source: PLoS One. 2021 Feb 5;16(2):e0246402. doi: 10.1371/journal.pone.0246402 (PMC7864402; doi:10.1371/journal.pone.0246402)

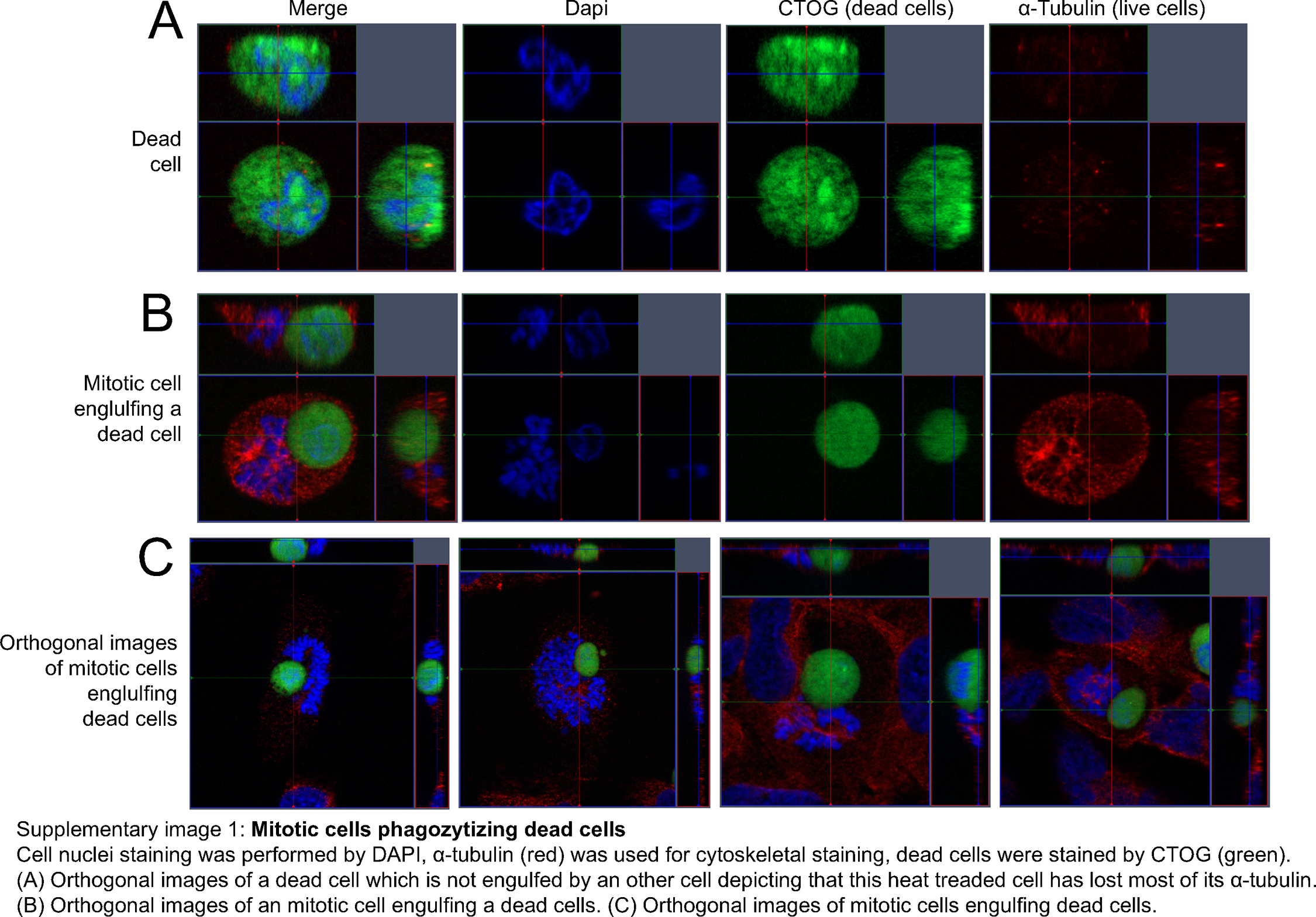

Supplement: S1 Fig — Cell nuclei staining was performed by DAPI, α-tubulin (red) was used for cytoskeletal staining, dead cells were stained by CTOG (green). (A) Orthogonal images of a dead cell which is not engulfed by an other cell depicting that this heat treaded cell has lost most of its α-tubulin. (B) Orthogonal images of an mitotic cell engulfing a dead cells. (C) Orthogonal images of mitotic cells engulfing dead cells. (TIF) [file pone.0246402.s001.tif]
